# Ferrogels Ultrasonography for Biomedical Applications

**DOI:** 10.3390/s19183959

**Published:** 2019-09-13

**Authors:** Felix A. Blyakhman, Sergey Yu Sokolov, Alexander P. Safronov, Olga A. Dinislamova, Tatyana F. Shklyar, Andrey Yu Zubarev, Galina V. Kurlyandskaya

**Affiliations:** 1 Ural State Medical University, 620028 Ekaterinburg, Russia; feliks.blyakhman@urfu.ru (F.A.B.); sergey.sokolov@urfu.ru (S.Y.S.); o_dinislamova@rambler.ru (O.A.D.); t.f.shkliar@urfu.ru (T.F.S.); 2 Institute of Natural Sciences and Mathematics Ural Federal University, 620002 Ekaterinburg, Russia; safronov@iep.uran.ru (A.P.S.); A.J.Zubarev@urfu.ru (A.Y.Z.); 3 Institute of Electrophysics, Ural Division RAS, 620016 Ekaterinburg, Russia; 4 M.N. Mikheev Institute of Metal Physics of the Ural Branch of the Russian Academy of Sciences, 620990 Ekaterinburg, Russia; 5 Departamento de Electricidad y Electrónica and BCMaterials, Universidad del País Vasco UPV/EHU, 48080 Bilbao, Spain

**Keywords:** magnetic nanoparticles, ferrogels, medical ultrasound, sonography, biomedical applications

## Abstract

Ferrogels (FG) are magnetic composites that are widely used in the area of biomedical engineering and biosensing. In this work, ferrogels with different concentrations of magnetic nanoparticles (MNPs) were synthesized by the radical polymerization of acrylamide in stabilized aqueous ferrofluid. FG samples were prepared in various shapes that are suitable for different characterization techniques. Thin cylindrical samples were used to simulate the case of targeted drug delivery test through blood vessels. Samples of larger size that were in the shape of cylindrical plates were used for the evaluation of the FG applicability as substitutes for damaged structures, such as bone or cartilage tissues. Regardless of the shape of the samples and the conditions of their location, the boundaries of FG were confidently visualized over the entire range of concentrations of MNPs while using medical ultrasound. The amplitude of the reflected echo signal was higher for the higher concentration of MNPs in the gel. This result was not related to the influence of the MNPs on the intensity of the reflected echo signal directly, since the wavelength of the ultrasonic effect used is much larger than the particle size. Qualitative theoretical model for the understanding of the experimental results was proposed while taking into account the concept that at the acoustic oscillations of the hydrogel, the macromolecular net, and water in the gel porous structure experience the viscous Stocks-like interaction.

## 1. Introduction

Hydrogels are soft materials that are widely used in the area of biomedical applications [1]. Ferrogels (FG) are composites that contain a polymer swollen in a solvent and filled with nano- or micro-sized magnetic particles [2,3,4]. In particular, FG based on polyacrylamide (PAA) hydrogel with magnetic nanoparticles (MNPs) of iron oxides are the most studied and sought-after material for a wide range of biomedical applications, including magnetic biosensors, drug delivery, and regenerative medicine [5,6]. Different studies had established the fact that the elastic properties of PAA ferrogels can significantly vary, depending on the details of synthesis conditions, as well as change their mechanical characteristics in response to an external magnetic field [4,7,8]. It is also known that polyacrylamide ferrogels have low toxicity and good compatibility to living cells [9,10]. Based on this advantage, PAA ferrogels have been used as substrates (scaffolds) for cell culturing for the needs of cellular technologies and tissue engineering [11].

Ferrogel scaffolds offer different directions of the research and applications. For example, the intensity of ultrasound that is reflected at a ferrogel/water interface is determined by the difference between the acoustic impedances of the two materials. The effect of magnetic nanoparticles on the ultrasonic parameters (also acoustic impedance) of gel phantoms has been previously shown [12]. The values of the impedance increased after the addition of nanoparticles, so the phantoms with magnetic nanoparticles exhibited increased echogenicity, owing to the significant number of scatters [13].

In addition, the MNPs forming the part of the PAA gels have high magnetic responses, often sufficient for the control of the movement of FG based micro-objects while using an external magnetic field [14]. This feature opens up prospects for the use of ferrogels for magnetic biosensor applications, regenerative medicine, or targeted drug delivery or controlled release of drugs [15,16,17]. The above-mentioned applications are associated with the solution of a number of problems, including the task of reliable visualization of FG in a living organism.

In fact, the scientific community has already moved from the concept of biomedical applications of magnetic nanoparticles toward the understanding of ferrogel models, which are much closer to realistic applications in which MNPs are distributed in living tissue. Very often, such distribution reflects the biological properties of the natural tissue. What is especially important, aggregation features can be conditioned by irregularly structured tissue with the disease-affected morphology [18]. The development of a new technique for detecting of ferrogel scaffolds in blood vessels while using medical ultrasound is highly desired. There is a strong request for FG use in vivo as a prototype of magnetically controlled platforms for targeted delivery of cell implants and drug substrates through arteries, as well as positioning indicators for low invasive surgery.

In the work, the features of ultrasonic location of samples of PAA based ferrogels with a variation of the concentration of Fe_2_O_3_ MNPs of iron oxide in a wide range are considered for FG scaffold of different shape. A theoretical interpretation of the experimental results is given.

## 2. Experimental

### 2.1. Gel and Ferrogel Synthesis and Characterization

**Figure 1:**
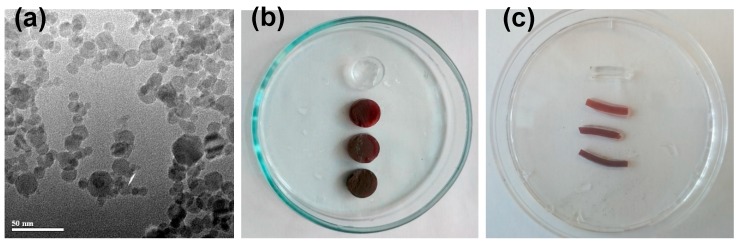
Transmission electron microscopy (TEM) image of laser target evaporation (LTE) iron oxide magnetic nanoparticles (MNPs) used for ferrogel synthesis (**a**). General view of gel and ferrogel samples from batch #1 (**b**) and batch #2 (**c**). See explanation in the text.

Ferrogels with varying concentration of iron oxide magnetic nanoparticles were synthesized by three-dimensional (3D) radical polymerization of acrylamide in stabilized aqueous ferrofluid. First, electrostatically stabilized ferrofluid was prepared with stock concentration 5.1% of MNPs by weight. Fe_2_O_3_ MNPs (Figure 1a) were synthesized by the laser target evaporation (LTE) method while using commercial magnetite (Fe_3_O_4_) (Alfa Aesar, Ward Hill, MA, USA) as a precursor.

Technological details of the LTE method were previously described in earlier studies [19,20].

Stock ferrofluid for the synthesis of FGs was prepared by the ultrasound dispersion of maghemite MNPs in 5 mM solution of sodium citrate, taken as electrostatic dispersant. Ferrofluid was centrifuged for 5 min. at 10,000 rpm to eliminate large aggregates. The number averaged diameter of particles in ferrofluid determined by dynamic light scattering (Brookhaven ZetaPlus, Brookhaven Instruments Corp., Holtville, NY, USA) was found to be close to 33 nm. The resulted concentration of ferrofluid (5.1%) was determined as the dry weight residue after evaporation at 90 °C in an oven.

The stock ferrofluid was then diluted and used as a medium for the synthesis of FGs with varying content of MNPs. Monomer—acrylamide (AppliChem)—was used in 1.6 M concentration, a cross-linking agent—dimethylacrylamide (Merck)—was taken in 1/100 molar ratio to the monomer. Ammonium persulfate (APS) was used as an initiator in 3 mM concentration and the polymerization was performed at 70 °C for 2 h. N,N,N’,N’-tetramethylethylene diamine (TEMED) (SigmaAldrich Inc. St. Louis MO, USA) in 6 mM concentration was used as a catalyst. After the synthesis, FGs were extensively washed in distilled water with daily water renewal for two weeks. During this period, FGs swelled to equilibrium. The equilibrium water uptake (*Q*) was determined by gravimetry while using the equation:

(1) $$Q=\frac{{m\;-\;m}_{0}}{m_{0}},$$

with *m* denoting the weight of a swollen gel and *m*_0_ denoting the weight of a residue after drying a gel in an oven at 70 °C. The values of *Q* were used for the calculation of the actual content of iron oxide MNPs in a swollen ferrogel (*ω*), according to the equation:

(2) $$\omega =\frac{\gamma }{Q+1},$$

with *γ* denoting the weight fraction of iron oxide MNPs in the dry residue of ferrogel. The value of *γ* was determined based on the composition of the reaction mixture in the synthesis.

Two batches of FG samples were synthesized differently in their shape. Ferrogels of batch #1 were synthesized in cylindrical polyethylene moulds 8.5 mm in diameter and 50 mm in height. These gels were used for ultrasonography studies in water, and further on used for the mechanical testing experiments. Therefore, they were cut into cylindrical plaques of the size of approximately 5 mm (in height). The diameter of cylinders was approximately 13 mm conditioned by the equilibrium swelling of a gel in water after the synthesis. FGs of batch #1 contained 0.00, 0.33, 0.64, and 1.34% of maghemite iron oxide MNPs by weight. Further on, they are denoted as FG1-0, FG1-1, FG1-2, and FG1-3 samples. A general view of FG samples of batch #1 is given in Figure 1b.

**Figure 2:**
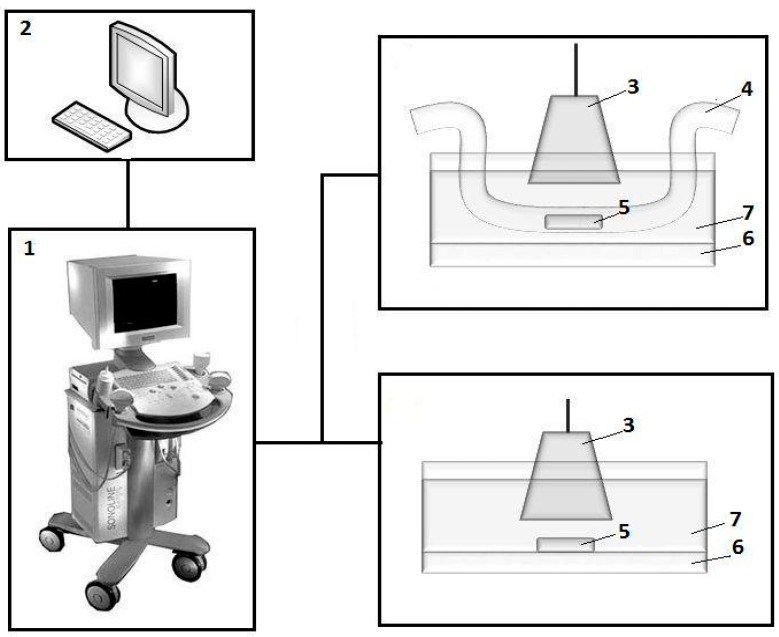
Scheme of the experimental setup to determine the echogenic properties of gels/ferrogels: 1—ultrasonic apparatus; 2—personal computer; 3—sensor of ultrasonic apparatus; 4—silicone tube with water; 5—sample of the gel/ferrogel; 6—soft pad; and, 7—cuvette with water. See also explanation in the text.

Ferrogels of batch #2 were synthesized in capillary polyethylene moulds 1.7 mm in diameter and 20 mm in height. These gels were used for ultrasonography studies in the configuration modelling the blood vessel (tube). These samples were cut in small cylinders of approximately 6 mm in length. The diameter of the cylinders was approximately 2 mm, provided by the equilibrium swelling of a gel in water after the synthesis. Ferrogels of batch #2 contained 0.00, 0.55, 0.98, and 1.45% of iron oxide MNPs by weight. Further on, they are denoted as FG2-0, FG2-1, FG2-2, and FG2-3 samples. Figure 2 provides a general view of ferrogel samples of batch #2. The content of iron oxide MNPs in ferrogels of batch#1 and batch#2 slightly differed due to the variation in the composition of the reaction mixtures. In the context of proposed study, we did not set out to synthesize samples with exact the same concentration of particles in two batches.

Synthesized ferrogels were uniformly colored and transparent (see Figure 1b,c). Visually, their appearance was similar to that of the precursor ferrofluid that was taken for the synthesis. No signs of turbidity were noticeable (including observations at the level of optical microscopy). Thus, it might be considered that there was no substantial aggregation of iron oxide MNPs in the synthesis of ferrogels and the distribution of MNPs in ferrogels was uniform, as in the precursor ferrofluid.

The elastic properties of the gels were evaluated by mechanical testing of the samples as described elsewhere [6,8,10]. Briefly, stepped strains for compression of up to 20% of the initial length were set to samples of gels while using a linear motor with a step of 1–2%. As a result, the “stress- strain” (σ-ε) dependencies were obtained, from the linear part of which the Young’s modulus was determined for each one of the samples. For these series of experiments, samples of gels from the batch #1 (cylinders with a diameter of 13 mm and a height of 5 mm) were used (Figure 1b).

The magnetic hysteresis loops M(H) of the MNPs of iron oxide, gels, and ferrogels were measured by vibrating sample magnetometer (VSM, Faraday magnetometer of laboratory design). The maximum value of the magnetization was obtained for the external field of 1.5 kOe. For simplicity, it was denominated as saturation magnetization (Ms). The coercivity (Hc) was also estimated from the shape of the M(H) hysteresis loop. Thermomagnetic zero field cool–field cool curve (ZFC-FC) was measured for air dry MNPs following standard procedure for magnetic nanoparticles [19]. The external magnetic field for the ZFC-FC curve was as high as H = 100 Oe (it was applied for cooling and heating in the case of FC part of the curve and for heating only in the case of ZFC part of the curve).

### 2.2. Experimental Setup for the Gels’ Ultrasound Visualization

Figure 2 shows an experimental setup for the study of the echogenic properties of gels and ferrogels. Samples of gels from the first batch (Figure 1b) were placed at the bottom of a cuvette with distilled water.

Samples from the second batch (Figure 1c) were located inside the silicone tube with an inner diameter of 6 mm and a wall thickness of 3 mm. The tube served for modelling the blood vessel configuration. It was filled with distilled water.

Fluid flow was not considered at this moment. Samples of the gels were visualized while using a Sonoline Adara (Siemens, Munich, Germany) medical device with a SIEMENS 7.5L45s Prima/Adara linear sensor. Ultrasonic sensor was immersed into a 500 mL cuvette that was filled with water for providing an acoustic contact. A gasket of soft viscose absorbent fibers was placed at the bottom of the cuvette in order to avoid the contribution of reflecting ultrasonic signal from the bottom of the cuvette. The video output of the ultrasound unit was connected to a computer equipped with an AverTV Hybrid VolarHX video capture device.

The dynamic range of the ultrasonic device in the mode of reception of the reflected oscillations was 66 dB, working frequency of 10 MHz, and the wavelength of 0.15 mm. In the experiments that were carried out within the framework of the corresponding batch of samples, the settings of the ultrasonic apparatus (radiation power, amplification, dynamic range, depth of visualization, etc.) were kept constant. The image of samples in two-dimensional (2D) mode in gray scale was recorded in a video file with a duration of several seconds with a frame rate of 25 frames per second, and the frame size is 720 pixels × 576 pixels.

Special software was developed to quantify the brightness of the image in various areas. It allowed to measure the brightness in the vicinity of a point specified by the user. In the experiment, the brightness of the image at the gel-water interface was measured and estimated. As a rule, the border thickness important for imaging was as high as 4–5 pixels. On this basis, the size of the area for assessing the brightness was limited to a square of 3 pixels × 3 pixels, which corresponds to linear dimensions of about 0.2 mm × 0.2 mm. For each sample, the measurements were carried out along the entire boundary of the gel. The number of measurements was at least 15 along the entire length of the sample. In each studied area, the minimum, maximum, and average image brightness were evaluated. The brightness was characterized in arbitrary units and it ranged from 0 (black) to 255 (white). For each type of FG, the average value of the maximum and average brightness, as well as the limits of the confidence interval at a significance level of *p* = 0.05, were calculated. In addition, in all experiments, 20 pixels × 20 pixels square image was used to estimate the background, i.e., the brightness of the image area where the water was located. The maximum, minimum and average background brightness in all cases varied insignificantly. Therefore, finally, the adjustment that was associated with changes in the brightness of the background was not carried out.

## 3. Results and Discussion

### 3.1. Structural and Magnetic Characterization of Nanoparticles and Ferrogels

Figure 1a shows an example of transmission electron microscopy (TEM) image of iron oxide MNPs (JEOL JEM2100, Tokyo, Japan). The majority of MNPs have spherical shape. Very few of them contain hexagonal corners. Particle size distribution (PSD) of MNPs was lognormal with the median d_0_ = 11.7 nm and the logarithmic dispersion σ = 0.423, as determined by the graphical analysis of 2150 TEM images. According to PSD, the average diameter of 93% of MNPs (by weight) fits a 5–40 nm range. According to X-ray diffraction (Bruker D8 Discover, Billerica, MA, USA), the crystalline structure of MNPs was an inverse spinel with space group Fd3m. The lattice parameters corresponded to maghemite (Fe_2_O_3_). The oxidation number +3 of Fe ions in the chemical composition of MNPs was confirmed by Ox/Red titration (TitroLine, Schott Instruments).

Measurements of the saturation magnetization of as-prepared air dried MNPs showed that their average size and defined composition (γ-Fe_2_O_3_) are quite consistent with each other: M_s_ ≈ 40 emu/g. Detailed discussion on the magnetic structure of “core-shell” LTE MNPs can be found elsewhere [19,20].

**Figure 3:**
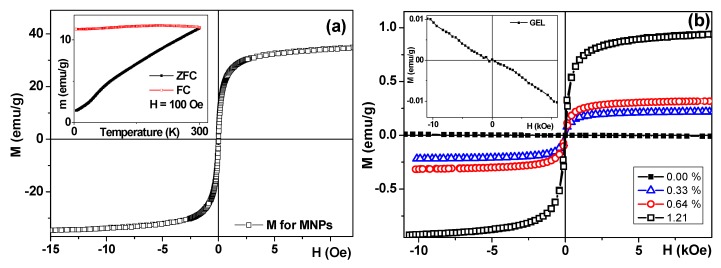
Magnetic hysteresis loop of air-dry nanoparticles measured at room temperature; inset shows thermomagnetic zero field cool–field cool curve (ZFC-FC) curve (**a**). Hysteresis loops for FG1-0, FG1-1, FG1-2, and FG1-3 samples measured at room temperature (**b**); inset shows diamagnetic response of the blank gel.

As before, pure gel without nanoparticles showed linear non-hysteretic diamagnetic response on the application of external magnetic field (Figure 3a). At the same time, the ferrogel’s M(H) loops had a typical S-shape with negligible coercivity for the small concentrations of MNPs (Figure 3b). The evolution of the Ms value as a function of the concentration of nanoparticles in the ferrogel shows linear dependence: the higher concentration, the higher the Ms (Figure 3b). The magnetization value for the FG scaffold in the applied magnetic field of certain strength is a very important parameter in a view of FGs applications for drug delivery and the controlled movement of micro-objects by magnetic field. For the highest concentration of 1.45 wt. % in the external field of 0.5 kOe (reasonably low an accessible for generation), the magnetic moment of the FG was as high as 0.5 emu/g. Taking into account the scaffold FG2-3 volume, one can obtain the magnetic moment of the sample in the 0.5 kOe external magnetic field: m = 0.9 × 10^−3^ emu.

### 3.2. Gels Elasticity

**Figure 4:**
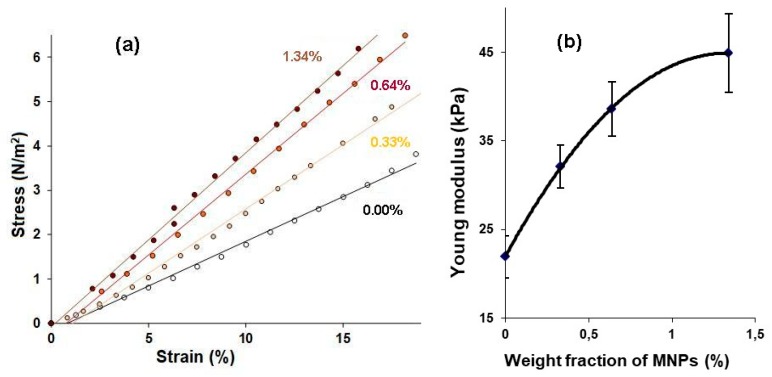
Example of the ‘stress-strain’ relationship for gels of batch #1 (**a**) and the dependence of Young’s modulus on the MNPs concentration in gels of batch #1 (**b**). Vertical bars reflect the confidence interval with p = 0.05. Following fit parameters were obtained for stress (σ). For concentration 0.00%: σ = 20.2ε − 0.2, R^2^ = 0.995. For concentration 0.33%: σ = 28.9ε − 0.3, R^2^ = 0.994. For concentration 0.64: σ = 36.4ε − 0.3, R^2^ = 0.997. For concentration 1.34: σ = 39.2ε − 0.1, R^2^ = 0.998.

Figure 4a shows a plot of ‘stress-strain’ relationship for one of the samples of the first batch of gels (0.00, 0.33, 0.64 and 1.34 wt.% of MNPs). It is seen that, at any fixed strain, the stress in the gel is the greater, the higher the concentration of MNPs. The caption contains the linear regression equations for each sample, where the value of *ε* corresponds to the value of Young’s modulus in kPa.

Figure 4b shows the dependence of the Young’s modulus on the concentration of MNPs in the gel/ferrogel for all the tested samples (six samples for each type of gel/ferrogel). First of all, it can be seen that the addition of MNPs to the PAA gel in the minimum concentration (0.33%) leads to a significant increase in the Young’s modulus of the composite material. Secondly, a gradual increase in the concentration of MNPs in ferrogel is accompanied by a further increase in its elasticity. The results that were obtained are in good agreement with the data of our earlier studies [6,8,11] and the findings of other authors [7,21].

### 3.3. Gel/water Boundary Echogenicity

**Figure 5:**
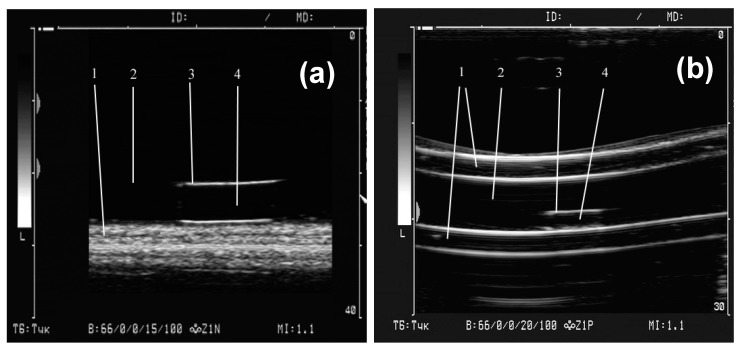
Examples of ferrogels samples visualization at the bottom of water cuvette (**a**) and inside a silicone tube filled with water (**b**). The distance from sensor to objects is about 20 mm. 1—soft pad (a) and wall of silicone tube (b); 2—water in cuvette (a) and in tube (b); 3—upper boundary of gel/water; 4—gel body.

Figure 5 shows an example of a single ultrasound image frame when scanning a gel sample positioned at the bottom of a cuvette (a) and inside a model vessel (b) (Figure 2). The images very clearly show the boundaries of the gel samples lining at the bottom of the cuvette and the walls of the tube with water. It is also seen that the highest echogenicity of the gels (i.e., the largest amplitude of the reflected ultrasonic vibrations, and, accordingly, the brightness of the image) corresponds not to the sample body, but to the interface between the gel surface and the water. In experiments with the first batch of gels, the visualization was performed with an ultrasound device amplification of 15 dB. Four samples were tested for each concentration of MNPs in the gel. The average value of the maximum brightness of the water in the cuvette in all tests of the gels was 34.1 ± 0.2 (n = 16).

**Figure 6:**
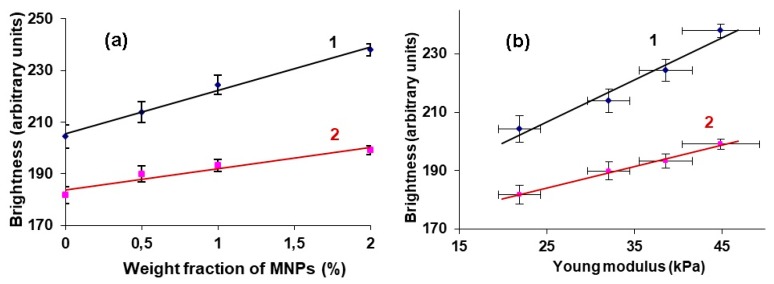
Dependences of maximal (1) and average brightness (2) at gel/water boundary on the MNPs concentration for samples of batch #1 (**a**). Vertical bars reflect the confidence interval with p = 0.05. Dependences of maximum (1) and average brightness (2) at gel/water boundary on Young’s modulus for samples of batch #1 (**b**). Vertical and horizontal bars reflect the confidence interval of relevant parameter with p = 0.05.

Figure 6a shows the dependence of the maximum and the average brightness at the gel/water interface on the concentration of MNPs in the gel. The graphs show the boundaries of confidence intervals at p = 0.05. It can be seen that, by increasing the weight fraction of the MNPs in the samples, both the maximum brightness (1) and the average brightness (2) of the echo reflected from the surface of the gels were increased. The data are well approximated by the linear regressions: (1) y = 16.783x + 205.4, R^2^ = 0.988; (2) y = 8.166x + 183.8, R^2^ = 0.931.

Figure 6b shows the dependences of the maximum and average brightness of the gel/water interface on the Young’s modulus of the samples. It can be seen that the echogenicity of the surface of the gels is directly related to the elastic properties of the tested materials. Moreover, the higher the stiffness of the samples, the more accurately the gels are visualized. Again, the data (Figure 6b) are well approximated by the linear regressions: (1) y = 1.529x + 169.45, R^2^ = 0.989; and, (2) y = 0.7666x + 165.63, R^2^ = 0.987.

In a series of experiments with the second batch of gels (samples were placed in a model “blood” vessel (Figure 1c and Figure 5b), the ultrasonic amplification of the apparatus was increased to 20 dB to compensate for the signal loss through the tube wall. Totally, seven samples were tested for each concentration of MNPs in the gel. The average brightness of the water inside the tube was 44.4 ± 0.2 (n = 28).

**Figure 7:**
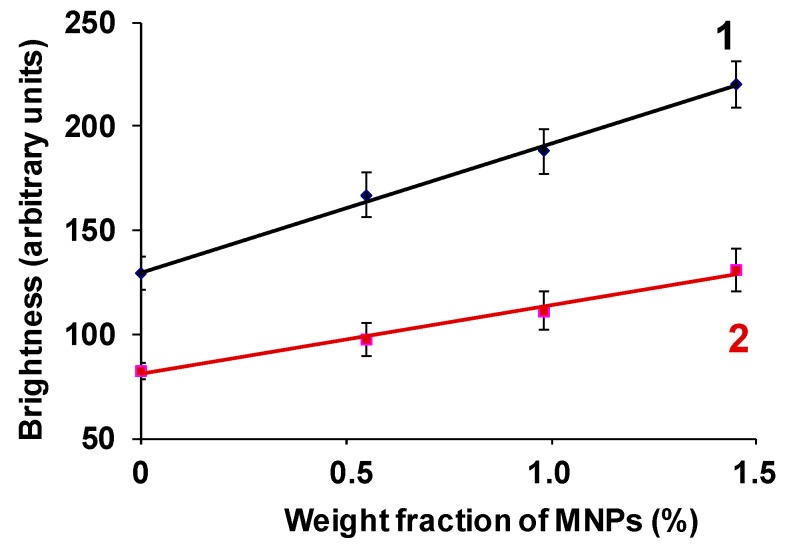
Dependences of maximum (1) and average brightness (2) at gel/water boundary on the MNPs concentration for samples of batch #2. Vertical bars reflect the confidence interval with *p* = 0.05.

Figure 7 shows a graph of the maximum and average brightness of the reflected echo signal at the gel/water interface in a tube versus the concentration of MNP in the sample. The graphs show the boundaries of confidence intervals at p = 0.05. The data (Figure 7) are well approximated by the linear regressions: (1) y = 61.652x + 130.07, R^2^ = 0.996; (2) y = 31.9x + 81.6, R^2^ = 0.984. It can be seen that the echogenicity of the surface of the ferrogel significantly increases in comparison with the PAA gel, even with the minimum concentration of MNPs. At the maximum concentration of MNPs in ferrogel (1.45%), the brightness of the reflected echo signal from its boundary with water is approximately two times higher than for the baseline PAA of the sample. This conclusion is valid for both the use of maximum and average brightness as a measure of the echogenicity of the material. The result that was obtained for the samples of gels (the first batch) inside the model vessel is in full compliance with the test data of the echogenicity of the samples (second batch) in water (see Figure 6a).

It should be noted that the dependence of brightness at the gel/water interface on the concentration of MNPs is more pronounced for the samples in a model vessel than for gels that were placed in a cuvette filled with water.

We have studied the ultrasound reflection from the non-deformed sample in condition, which mimic the ferrogel in a blood vessel. It is important to mention that effect of the sample preliminary tension on the sound reflection is out of scope of this work. Figure 4, Figure 6 and Figure 7 demonstrate that the Young modulus and the brightness of the reflected signal both increase with the particle concentration. It allows for making simple comment on the relation between the Young’s modulus and the impedance and to conclude that the impedance increases with the modulus. Note that classical results of the acoustic theory demonstrate the increasing relation on the basis of the general considerations of the theory of continuous media.

## 4. Discussion

The experience of ultrasound imaging of such polymer soft materials as gels for medical purposes is well known. In particular, the results of the location of gel phantoms of internal organs and tissues were used for testing and calibration of ultrasound diagnostic equipment [22,23], as well as implants that are based on gels [24,25]. Here, the visualization of FG sample geometrical features was for the first time achieved by using the ultrasonic location method. While taking into account the fact that FGs are considered to be promising materials for medical applications, the study was performed by a standard ultrasound apparatus that is in use for medical purposes. Regardless the shape of the samples and the conditions of their location, the boundaries of ferrogels are confidently visualized while using medical ultrasound over the entire range of concentrations of MNPs.

An increase in the MNPs concentration in the PAA gel was accompanied not only by an increase in the echogenicity of the gel/water interface, but also by the elasticity of the samples. Moreover, we established a linear relationship between the brightness of the echo signal reflected from the boundary and the Young’s modulus of the FG (Figure 6b). This result was obtained for the samples from the first batch of gels, while visualizing them at the bottom of a container with water.

It should be mentioned that in a series of experiments with a model vessel, the elastic properties of FGs from the second batch were not determined due to the design of the equipment for mechanical properties evaluation. When the diameter of the samples was below 8 mm, the installation did not allow for correct setting of deformations in compression. However, all of the materials and procedures used for the synthesis of gels were the same. The only exception was the moulding of gels for relevant needs. This circumstance makes it possible to insure the possible connection between the echogenicity of the surface of the gels and the Young’s modulus of samples from the second series with a high degree of confidence. Below, we attempt to describe the relationship between the concentration of MNPs in the gel and its echogenicity at the interface with water theoretically.

In this part, we suggest some physical interpretation of the experimental results. It is based on the concept that, at the acoustic oscillations of the hydrogel, the macromolecular net and water in the gel porous experience the viscous Stocks-like interaction. Thus, in the first approximation, one can suppose that, in each small volume of the hydrogel, the net and water move with the same velocity and the hydrogel can be considered as a unite continuum. In the frame of this physical approximation, the coefficient *R* of the sound reflection on the border between hydrogel and water out of it, can be estimated, as follows [26]

(3) $$R={\left(\frac{c_{2}\rho_{2}-c_{1}\rho_{1}}{c_{2}\rho_{2}+c_{1}\rho_{1}}\right)}^{2}$$

Here, the Subscribes 1 and 2 correspond to the water out of the hydrogel and the hydrogel, considered as a continuous medium, respectively, *ρ* is mass density of the corresponding media.

The sound speed can be presented as in Ref. [26]

(4) $$c=\sqrt{\frac{K}{\rho }}$$

where *K* is the compression modulus of the media. Therefore, Equation (3) can be rewritten as form:

(5) $$R={\left(\frac{\sqrt{K_{2}\rho_{2}}-\sqrt{K_{1}\rho_{1}}}{\sqrt{K_{2}\rho_{2}}+\sqrt{K_{1}\rho_{1}}}\right)}^{2}$$

The hydrogel compression modulus *K*_2_ is determined by the modulus of the water, modulus of the macromolecular net, and modulus of the embedded particles (see, for example, general discussion in [27]. Since the volume concentration of the gel and particles in the hydrogel are low, in the first approximation *K*_2_ ≈ *K*_1_. The hydrogel mass density is more than the density of water, i.e., *ρ*_2_ > *ρ*_1_. Therefore, the inequality (*K_*2*_ρ*_2_)^1/2^ > (*K_*1*_ρ*_1_)^1/2^ is held and the coefficient *R* can have quite significant value, which is enough to provide the visible reflection signal (see Figure 4). The addition of the rigid particles increases both the modulus *K*_2_ of the hydrogel and its density *ρ*_2_. Obviously, in the range of small concentrations of the particles, coefficient *R* (defined for Equation (5)) should show linear dependence on the concentration (Figure 6a and Figure 7).

The suggested theoretical interpretation of the experimental results is rather qualitative. Since the ultrasound wavelength is much more than the size of the particles and heterogeneities, provoked by the particles, we have used classic considerations of the acoustics of continuous media. It appeared enough for, at least, principal explanation of the experimental results. Detailed experimental study of the particles distribution inside the hydrogel are required in order to achieve reliable quantitative description of the reflection effects at higher level of understanding.

Let us make short remark on possible future directions of ultrasound location research. As it was mentioned in the introduction, magnetically controlled platforms for targeted delivery of cell implants and drug substrates through arteries, as well as positioning indicators for low invasive surgery, is a hot topic of the research and applications. Recently, we proposed using giant magnetoimpedance based multilayered sensitive element for the monitoring of FG scaffold position inside the blood vessel in regenerative medicine case of application [8,28].

Generally speaking, the goal to define the FG scaffold position can be completed either by magnetic or by ultrasound detection. What is most important is that both techniques can probably be used at a time or just one after another in two simple non-invasive tests. The concept of usage of different techniques for material characterization is well established in the nanomedicine for nanomaterials characterization [16]. Ferrogels offer a new opportunity for extending this concept on the non-invasive tests, keeping in mind not a material characterization, but rather complex diagnostic solution. Different techniques have their advantages and disadvantages and the application of multiple techniques can be viewed as additional advantage, especially keeping in mind than ultrasonic equipments are available for routine diagnostics.

## 5. Conclusions and Outlook

Samples of ferrogels with different concentrations of MNPs were synthesized in various forms to simulate different peculiarities of the visualization of sample location for future development in the area of biomedical applications. In particular, thin cylindrical samples were used to simulate the situation in which ferrogels can be used as platforms for targeted drug delivery or cell cultures through blood vessels. Samples of larger size in the form of cylindrical plates were used in order to simulate cell grafts that are based on ferrogel, intended for use as substitutes for damaged structures, such as bone or cartilage tissues.

We found that, regardless of the shape of the samples and the conditions of their location while using medical ultrasound, the boundaries of ferrogels are confidently visualized over the entire range of concentrations of MNPs. Moreover, the intensity of the reflected echo was greater, the greater the concentration of MNPs in the gel. Obviously, the result that was obtained is not related to the influence of the MNPs directly on the intensity of the reflected echo signal, since the wavelength of the ultrasonic effect used is much larger than the particle size. Therefore, we can point out the indirect effect of MNPs on the echogenicity of ferrogels.

Qualitative theoretical interpretation of the experimental results was proposed while taking into account the concept that, at the acoustic oscillations of the hydrogel, the macromolecular net and water in the gel porous experience the viscous Stocks-like interaction.
